# Assessment of mechanical characteristics of polyetheretherketone as orthodontic fixed lingual retainers

**DOI:** 10.1016/j.jds.2023.05.026

**Published:** 2023-06-08

**Authors:** Pyi Phyo Win, Daniel De-Shing Chen, Bolormaa Sainbayar, Tzu-Yu Peng, Johnson Hsin-Chung Cheng

**Affiliations:** a School of Dentistry, College of Oral Medicine, Taipei Medical University, Taipei, Taiwan; b Division of Orthodontics, Department of Dentistry, Taipei Medical University Hospital, Taipei, Taiwan; c School of Dentistry, Mongolian National University of Medical Sciences, Ulaanbaatar City, Mongolia

**Keywords:** Polyetheretherketone, Orthodontic, Fixed lingual retainer, Shape optimization, Bending test

## Abstract

**Background/purpose:**

Polyetheretherketone (PEEK) is known for its strength, flexibility, biocompatibility, and potential as a replacement for metals in dental appliances; however, uncertainty remains about the mechanical characteristics and dimensions of PEEK-made orthodontic fixed lingual retainers (FLRs). This study aimed to determine the optimal shape of PEEK-made orthodontic FLRs using the finite element method (FEM) and the three-point bending test (TPBT).

**Materials and methods:**

Seventy-five three-dimensional PEEK rod-shaped models were created, which included five thicknesses (0.4, 0.6, 0.8, 1.0, and 1.2 mm), five widths (0.7, 0.9, 1.1, 1.3, and 1.5 mm), and three cross-sectional shapes (rectangular, oval, and hemielliptical). A 0.9-mm (0.036-inch) stainless steel wire (SSW) was used as a control and the FEM was used to determine six optimal dimensions among the PEEK models. The selected models were then fabricated and subjected, along with the SSW, to the experimental TPBT to assess their mechanical responses against lingual and biting pressures.

**Results:**

The FEM analysis revealed that Von Mises stresses on the PEEK models decreased with an increase in width and thickness. Six optimal shapes of PEEK models were chosen based on acceptable lingual and biting stresses as well as patient comfort compared to the SSW. Furthermore, PEEK models showed significantly lower deformation during the 3.1-mm deflection test than did the SSW, while no notable differences were observed among different sizes of PEEK models. The hemielliptical PEEK model with a thickness of 1.0 mm and width of 1.5 mm was found to be mechanically robust enough to withstand lingual forces, while none of the PEEK models, including the SSW, were able to resist biting forces.

**Conclusion:**

Within the limitations of this *in vitro* study, PEEK-made orthodontic FLRs with a hemielliptical cross-sectional shape and a thickness-to-width ratio of 1.0:1.5 would be suitable for use as orthodontic FLRs.

## Introduction

Retention is considered crucial in avoiding orthodontic relapse and maintaining successful treatment outcomes, as a previous study reported significant differences between a non-retention group and a retention group during a 1-year follow-up.^1^ Fixed lingual retainers (FLR), first introduced by Rupert W. Knierim in 1973, are the most commonly used method by orthodontists to achieve retention.^2^ Metal wire and fiber-reinforced composite (FRC) materials are widely used by orthodontists in current dental practice to make retainers.^3^, ^4^, ^5^ Metal wires have limitations, including poor aesthetics with the risk of metal allergies and image distortion when using magnetic resonance imaging.^6^, ^7^, ^8^ Additionally, when metal wires are not passively adapted to the teeth or when thin wires fracture, they can cause undesirable tooth movement.^4^^,^^5^ FRCs were developed to be esthetically pleasing,^9^ but they are less durable,^10^ and the bonding failure rate is higher than multistranded stainless-steel wire.^11^ Recently, for better accuracy, individualized nickel-titanium wires^12^ and zirconia^13^ manufacturing using computer-aided design and manufacturing (CAD/CAM) were tested. However, the use of nickel-titanium wires is limited by their metal color, and patients with metal sensitivities might not tolerate them; additionally, the flexibility of zirconia for physiological tooth movements is still questionable.

With continued advancements in material sciences, dentists are increasingly turning their attention to the high-performance polymer material known as polyetheretherketone (PEEK). This is due to its excellent mechanical strength, good stability against high temperatures, high corrosion resistance, biocompatibility, and high elastic modulus (ranging 3.6–4.8 GPa) which is closer to that of human cortical bone (14 GPa) compared to metals (110–130 GPa) and zirconia (205 GPa).^14^^,^^15^ Additionally, PEEK has comparable or lower plaque affinity than zirconia, which is already superior to titanium.^16^ Furthermore, PEEK is radiolucent, making it possible to visualize underlying structures, in contrast to metal and zirconia, which are radiopaque.^17^ By utilizing CAD/CAM fabrication, PEEK can achieve true passive adaptation,^18^^,^^19^ while in recent years, its mechanical strength, stress resistance, and esthetics were improved by incorporating fibers or ceramics.^20^ PEEK is considered a promising material for FLRs due to its appropriate characteristics. Despite previous studies examining the use of PEEK for FLRs, research investigating optimal designs for this appliance is currently lacking.

The finite element method (FEM) is an engineering simulation program used to analyze the stress and deformations of complex structures that could reflect the real-world actual result. With the development of powerful computers and improvement of the software, FEM is now widely used in different types of professions including biomedical research.^21^ It was first introduced to dentistry in the 1970s to study the load distribution of our human teeth.^22^

The objective of this study was to evaluate the feasibility of utilizing PEEK as an FLR by testing its optimized design and mechanical properties using FEM and a three-point bending test (TPBT) and comparing it to stainless-steel wire (SSW).

## Materials and methods

### Three-dimensional model design and build up

**Figure 1:**
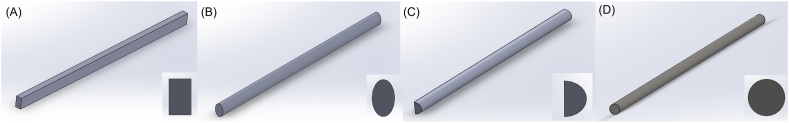
3D PEEK samples and 0.9-mm stainless steel wire (SSW). Small images are zoomed-in diagrams of each cross-sectional shape. (A) Rectangular, (B) oval, (C) hemielliptical, and (D) SSW.

Computer-aided-designed software (SolidWorks corp., Dassault Systemes, Waltham, MA, USA) was used to construct 30.0-mm rod-shaped three-dimensional (3D) PEEK test models with different widths, thicknesses, and cross-sectional shapes. The models were classified into five groups based on the widths, and each group was subdivided depending on the thickness. Later, each of the models was further produced into three shapes including rectangular, oval, and hemielliptical cross-sections (Fig. 1). Overall, there were 75 differently shaped 3D models in this study (Table 1). A 304 alloy 0.9-mm (0.036-inch) SSW was used as a control.

**Table 1:** Abbreviations of the grouping of test samples according to their different dimensions.

Width (mm)	Thickness (mm)				
	0.4	0.6	0.8	1.0	1.2
0.7	A1	A2	A3	A4	A5
0.9	B1	B2	B3	B4	B5
1.1	C1	C2	C3	C4	C5
1.3	D1	D2	D3	D4	D5
1.5	E1	E2	E3	E4	E5

Each sample was built in three cross-sectional shapes: rectangular, oval, and hemispherical. Width, the dimension that lies on the lingual surface of the tooth; thickness, the dimension that protruds toward the tongue.

### Shape optimization

The FEM was performed using simulation software (SolidWorks corp.) to conduct shape optimization. A fine high-quality mesh was prepared to achieve more-accurate numerical data along the nodes after stimulation. The material is known to be linearly elastic, homogeneous, and isotopic. The properties of each material used in the FEM were mentioned in Table 2. Each simulation was done under a non-bonded stage. The acceptable Von Mises stress of PEEK for an FLR was calculated in relation to our standard steel retainer control using the following formula:

$$M_{x}PEEK=\frac{M_{x}SS}{{YS}_{ss}}\times {YS}_{PEEK}$$

where Mx is the maximum acceptable Von Mises stress, YS is the yield stress, SS is stainless steel, YS of PEEK (YS_PEEK_) is 110 MPa, and YS of 304 alloy stainless steel (YS_SS_) is 207 MPa.

**Table 2:** Specification of polyetheretherketone (PEEK) and stainless steel used in the finite element method.

Property	PEEK (BioHPP)	304 alloy stainless steel control
Elastic modulus	4,800 MPa	190,000 MPa
Poisson's ratio	0.40	0.29
Mass density	1,400 kg/m^3^	8,000 kg/m^3^
Tensile strength	110 MPa	517 MPa
Yield stress	110 MPa	207 MPa

**Figure 2:**
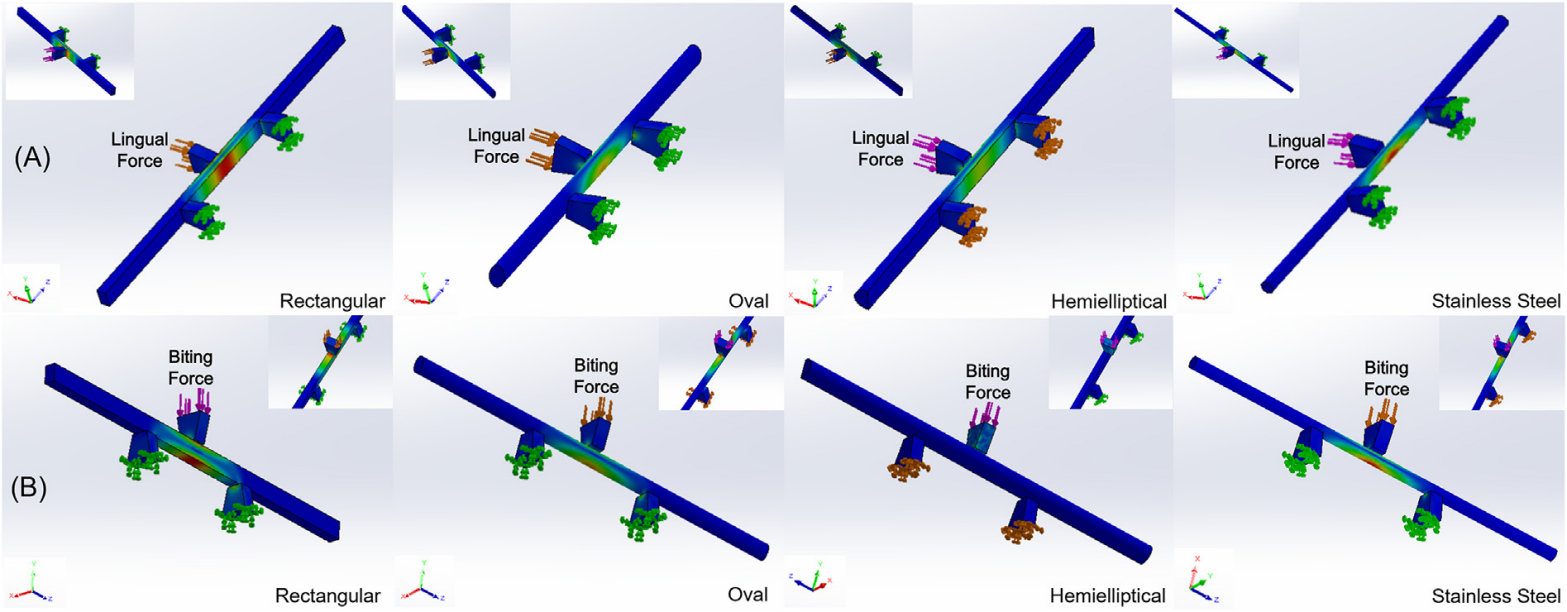
Representative Von Mises stresses (MPa) and distributions in the finite element model. Under stimulation of the lingual force (A) and biting force (B) tests, the small photos at the upper corner show the areas where the force was applied.

The maximum Von Mises stresses that occurred at the area of forces applied were recorded for comparison. All models were initially subjected to anteroposterior bending (10, 15, and 20 N)^23^^,^^24^ to simulate lip and lingual forces, and any models with unacceptable Von Mises stresses were eliminated (Fig. 2). The remaining models were then tested for resistance against vertical biting forces in the incisor region (225, 250, and 275 N)^25^^,^^26^, and two optimized designs from each cross-sectional shape were chosen based on Von Mises stress results and thickness considerations. Eventually, the six selected dimensions were fabricated into mechanical test specimens.

### Production of test samples

To produce mechanical test specimens, PEEK discs (BioHPP; Bredent GmbH & Co. KG, Senden, Germany) and a dental milling machine (DGSHAPE DWX-52DCI; Roland DG Corp., Shizuoka, Japan) were utilized. Twelve specimens of each dimension were prepared for an experimental test where six of them were for the lingual force test and the other six were for the biting force test. Before proceeding to the mechanical TPBT, all milled specimens were measured with digital calipers to calculate and check the precision and accuracy of the specimens.

### Experimental three-point bending test

The TPBT was conducted using a universal testing machine (JSV-H1000; Japan Instrumentation System Co., Ltd., Nara, Japan). Two supporting fulcrums were placed at 10.0 mm from each side of the edge, a crosshead rate of 1.0 mm/min was applied to the center of the models, and each model was pressed until a deflection of 3.1 mm was reached according to the International Organization for Standardization (ISO 15841) which is a system used to examine orthodontic wires. During the process, the maximum forces of each specimen were recorded, and the amount of deformation that appeared in each model was measured under an optical microscope (BA210; Motic Medical Diagnostic Systems, Co., Ltd., Xiamen, China) and recorded. Then, the mean values of each sample were compared to the SSW in relation to estimated lingual and biting forces. Finally, one dimension of the PEEK specimen which had appropriate durability and flexibility was determined as the optimal design suitable for use as an FLR.

### Statistical analysis

Average measurements, standard deviations (SDs), relative SDs (precision), and percent errors (accuracy) of all fabricated specimens were calculated. Results of shape optimization were recorded, and correlations of the width and thickness with maximum stress were obtained via Pearson correlations. Data comparisons between groups were conducted with a one-way analysis of variance (ANOVA) test. All of the above statistical analyses were carried out using standard statistical software (IBM SPSS version 19.0, Armonk, NY, USA).

## Results

### Shape optimization

**Figure 3:**
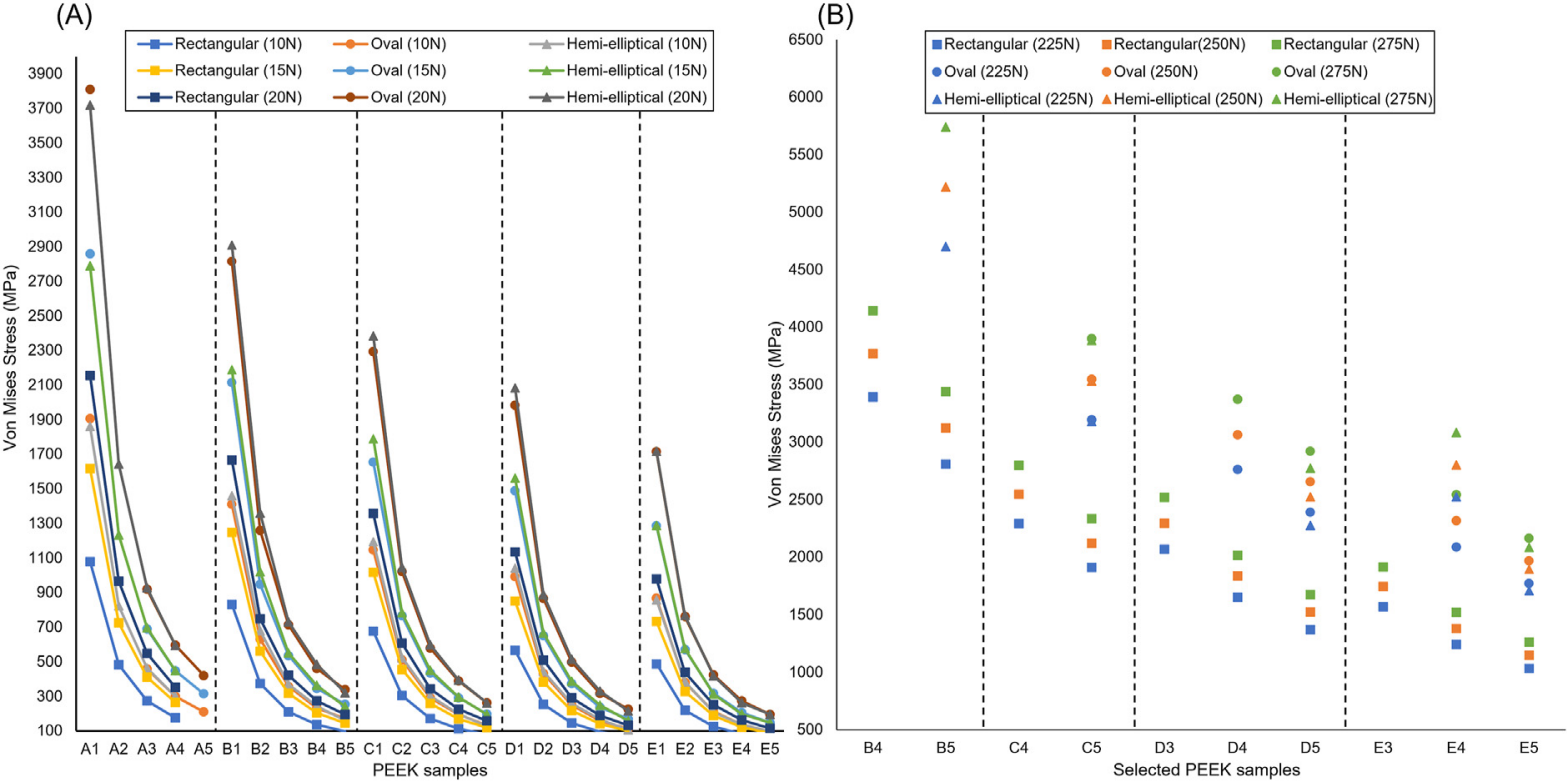
Estimated (A) lingual pressures (10/15/20 N) and (B) biting pressures in the incisor region (225/250/275 N) and their resulting Von Mises stresses (MPa) on each PEEK sample.

After calculating the allowable stresses on the 3D PEEK models under lingual forces, it was seen that the Von Mises stresses should be below 161.72, 239.66, and 318.75 MPa with 10, 15, and 20 N, respectively. After filtering out samples based on these values, 20 designs were selected. Ten dimensions from the rectangular shape (B4, B5, C4, C5, D3, D4, D5, E3, E4, and E5), five dimensions from the oval shape (C5, D4, D5, E4, and E5), and five dimensions from the hemielliptical shape (B5, C5, D5, E4, and E5) were found to be suitable for further bite force examination (Fig. 3A). Thicknesses of the samples showed a strong negative correlation with lingual pressure stresses, while the width of the samples showed a weak negative correlation with lingual pressure stresses (Table 3). In addition, acceptable Von Mises stresses for the 3D PEEK models were 3443.53, 3820.61, and 4197.58 MPa under biting forces of 225, 250, and 275 N, respectively. It was shown that all the models, except B5-hemielliptical, had stresses below the acceptable limit (Fig. 3B); meanwhile, the width had a strong negative correlation with biting force stresses, while thickness did not show a significant correlation (Table 3). Eventually, based on their strength and comfort, D3 and E3 with a rectangular shape, D4 and E4 with an oval shape, and C5 and E4 with a hemielliptical shape were selected for subsequent tests.

**Table 3:** Pearson correlations of width and thickness with lingual force and biting force.

	Width						Thickness					
	Lingual force			Biting force			Lingual force			Biting force		
	10 N	15 N	20 N	225 N	250 N	275 N	10 N	15 N	20 N	225 N	250 N	275 N
*r* value	−0.313	−0.313	−0.313	−0.750	−0.751	−0.751	−0.774	−0.774	−0.775	0.178	0.179	0.180
*P* value	0.008^a^	0.007^a^	0.008^a^	0.000^a^	0.000^a^	0.000^a^	0.000^a^	0.000^a^	0.000^a^	0.452	0.450	0.448

*r* value, Pearson correlation coefficient.

a Correlation was significant at the 0.05 level.

### Experimental mechanical test

**Figure 4:**
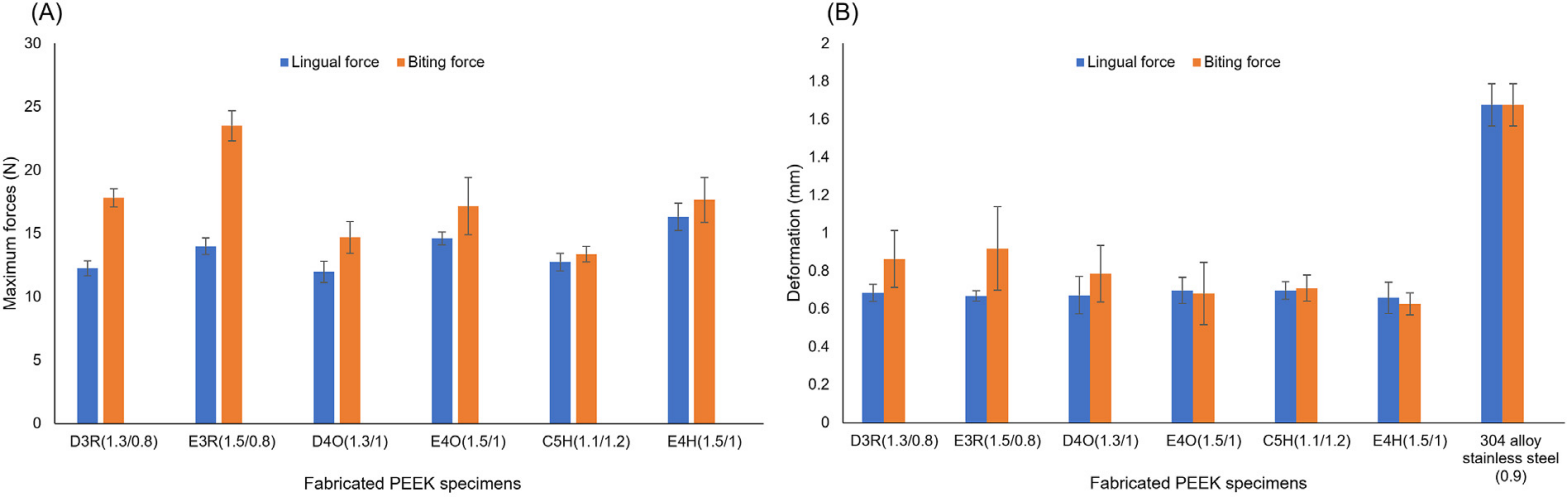
Maximum forces (A) and deformation (B) of each polyetheretherketone (PEEK) specimen recorded during the lingual and biting force tests.

Table 4 shows that the precision error was <1%, while the accuracy error was <4% compared to actual measurements. The smallest dimension for this test had a maximum error of 3.7%. The average maximum force for the SSW was 94.67 N in both the lingual and biting force tests. However, maximum forces for PEEK specimens differed depending on the direction of the applied force (Fig. 4A).

**Table 4:** Measurement confirmation of all fabricated polyetheretherketone (PEEK) specimens.

Groups	Exact measurements (L/W/T) (mm)	Actual measurements			Relative SD (%) (L/W/T)	Percent error (%) (L/W/T)
		L (mm)	W (mm)	T (mm)		
D3R	30.0/1.3/0.8	30.282 ± 0.140	1.303 ± 0.008	0.829 ± 0.006	0.5/0.6/0.7	0.9/0.2/3.7
E3R	30.0/1.5/0.8	30.293 ± 0.097	1.515 ± 0.005	0.825 ± 0.006	0.3/0.3/0.7	1.0/1.0/3.1
D4O	30.0/1.3/1.0	30.346 ± 0.097	1.290 ± 0.009	1.009 ± 0.006	0.3/0.7/0.6	1.2/0.8/0.9
E4O	30.0/1.5/1.0	30.307 ± 0.148	1.481 ± 0.006	1.014 ± 0.005	0.5/0.4/0.5	1.0/1.3/1.4
C5H	30.0/1.1/1.2	30.358 ± 0.143	1.096 ± 0.006	1.209 ± 0.0044	0.5/0.6/0.4	1.2/0.3/0.8
E4H	30.0/1.5/1.0	30.266 ± 0.079	1.503 ± 0.006	1.009 ± 0.0038	0.3/0.4/0.4	0.9/0.2/0.9

Groups were divided according to three different cross-sectional shapes of rectangular (R), oval (O), and hemielliptical (H). L, length; W, width; T, thickness of fabricated PEEK specimens. The values of exact and actual measurements are shown as the mean ± standard deviation (SD).

The maximum forces for lingual force testing ranged from 11.97 (D4-oval) to 16.32 N (E4-hemielliptical). Comparing these values to the estimated tongue pressure of 15 N,^21^, ^22^, ^23^ the SSW and E4-oval and E4-hemielliptical samples were able to withstand lingual pressure with respective forces of 94.67, 14.63, and 16.32 N. The SSW significantly differed from PEEK, and the E4-hemielliptical sample had the highest mean maximum force among all of the fabricated PEEK specimens. The second-highest dimension, E4-oval, showed no significant difference from E3-rectangular, while the remaining dimensions stayed under the maximum force of 14.00 N. Regarding the biting force, all tested specimens, including the SSW, were already bent to 3.1 mm without reaching our estimated biting force. Among PEEK specimens, maximum forces ranged 14.70 (D4-oval) to 23.52 N (E3-rectangular). Note that the E3-rectangular specimen was significantly stronger than other PEEK specimens, followed by the E4-hemielliptical, E4-oval, and D3-rectangular specimens as no significant difference was found among them.

Deformation of the test specimens after a deflection of up to 3.1 mm is shown in Fig. 4B. The SSW showed the highest deformation with an average deflection of 1.68 mm, and there was a significant difference between the SSW and PEEK samples. Among PEEK specimens, the highest deformation (0.70 mm) occurred in the E4-oval and C5-hemielliptical samples during bending in the anteroposterior direction. In the vertical direction, the E3-rectangular sample showed the highest deformation of 0.92 mm, followed by the D3-rectangular specimen (0.86 mm). However, no significant difference was found among permanent deformation values of fabricated PEEK specimens.

## Discussion

To maintain periodontal health and reduce stress at the bonding area, retainer wires must be flexible;^27^^,^^28^ however, using only canines for bonding has limitations, including bonding failure and the inability to prevent the tongue's protrusive effect on unbonded anterior teeth.^29^, ^30^, ^31^ To address these limitations, smaller, more-flexible multistranded wires have been used, but some studies suggested that these wires can cause undesirable tooth movements due to their twisting effect.^32^^,^^33^ In this study, PEEK was tested to evaluate the possibility of using it as an alternative material for orthodontic FLRs that can overcome the above drawbacks. Stout et al.^13^ found that a 0.9-mm SSW performed well as an FLR in an FEM, while a 0.5-mm wire failed the test. A 0.9-mm wire was successfully used in the past by bonding it only to the canine;^29^ thus, the 0.9-mm 304 alloy SSW was used as a control group in this study. PEEK wires were designed to be around the same size as the control 0.9-mm SSW, resulting in 25 different dimensions of samples. As to the cross-sectional shape, a broader width than thickness was chosen to provide better strength and more surface area for retention in a veneering method, while reducing foreign body sensitivity for the patient.^19^^,^^34^ Rectangular, oval, and hemielliptical shapes were selected, resulting in 75 3D PEEK samples included in this study (Table 1).

FEM was used for shape optimization as it is cost-effective and time-saving before fabricating actual specimens for mechanical tests.^35^^,^^36^ For the determination of force in the anteroposterior direction, the lingual force was taken since tongue pressure already covers the pressure from the lips. Dimensions that had smaller thicknesses with acceptable strength were chosen from this test. Exceptionally, hemielliptical shape models show some discrepancy because of the limitation of shape during model set-up for simulation. However, these differences would not affect our findings since the selection of the models in finite element method was done comparing the Von Mises stresses and the sizes within their own cross-sectional shape groups (Fig. 2). Notably, all of the selected dimensions of PEEK samples were relatively greater than those of the SSW which met the statement of Fitton et al.^37^ Moreover, among the selected models, rectangular cross-sectional shape possessed the smallest dimension of 0.8 mm compared to others, showing that rectangular shape was morphologically stronger than oval and hemielliptical shapes. According to the Pearson correlation (Table 3), the thickness resists the lingual force, while the width contributes to resisting biting and lingual forces. Choosing a larger width instead of thickness not only counters the biting force but also provides greater comfort and a larger surface area for adhesion and helps endure the lingual force.

The precision error was <1%, indicating consistency within PEEK specimens. The accuracy error was <4%, with the milling machine capable of fabricating PEEK models as small as 0.5 mm. The error increased to around 3.7% when fabricating the smaller 0.8 mm dimension (Table 4). Following the TPBT (Fig. 4), the maximum force recorded on the SSW for both lingual and biting force tests was 94.67 N, which was significantly higher than the estimated lingual force of 15 N in our study.^21^, ^22^, ^23^ Among PEEK specimens, three dimensions, E3-rectangular, E4-oval, and E4-hemielliptical, expressed the maximum force, which was close to our estimated lingual force value. The E4-hemielliptical (16.32 N) specimen had the significantly highest maximum load among all of the PEEK specimens. Sfondrini et al.^10^ tested a clinically used 0.028 × 0.008-inch flat metal wire, and the maximum load after deflection of 1.0 mm was 3.02 N, while the PEEK specimen mentioned above showed a higher maximum load of 12.38 N at the time of 1.0-mm deflection. Ohtonen et al.^38^ stated that there was no significant difference between the stiffness of the above 0.028 × 0.008-inch flat metal wire and five strands of 0.0215-inch SSW which was nominated by Zachrisson et al.^3^ as a gold standard orthodontic lingual retainer. Hence, the E4-hemielliptical specimen had a higher load deflection rate than clinically used SSW, making it resistant to deformation and preventing unwanted tooth movement or cracks within the composite.^39^ Biting force results indicated that neither the SSW nor fabricated PEEK specimens reached the maximum force of the estimated occlusal force at the incisal region (250 N).^24^, ^25^, ^26^ Serious mechanical deformation can occur when the force directly acts on the lingual fixed retainer. Patients should also be warned to be careful when biting with the anterior teeth. Since fixed retainers are bonded to the teeth, the shear bonding strength, which was not tested in this study, should also be considered for this vertical direction. Nonetheless, a previous article indicated that 0.0215-inch multistrand SSWs exerted forces of 1.01 and 1.59 N in the vertical and anteroposterior direction, respectively, during 0.2-mm deflection.^40^ Compared to E4-hemielliptical specimens, the vertical force test recorded 3.25 N, and the lingual force test recorded 2.70 N with 0.2-mm deflection. This dimension not only displayed the highest strength among all PEEK specimens during the lingual force test but also showed results similar to those of multistranded retainer wires used clinically.

In terms of deformation, the SSW showed a large deformation of 1.68 mm in both directions which was significantly higher than PEEK specimens. However, no significant difference was seen among PEEK specimens. In the lingual force test, the E4-oval and C5- hemielliptical specimens were more highly deformed than other dimensions, whereas the E3-rectangular specimen which was the strongest of all PEEK specimens during the biting force test showed the most permanent deformation in the same test. Sifakakis et al.^40^ stated that physiologic mobility values of mandibular incisors at 1.5 N are 0.06 mm in the anteroposterior direction and 0.2 mm in the vertical direction. E4-hemielliptical samples needed only 1.27 N for 0.06-mm anteroposterior displacement and 0.95 N for 0.02-mm vertical displacement, which indicated that this dimension was not only more flexible than the SSW but it was flexible enough to allow physiologic tooth movement.

The FLRs clinically follow a curved arch form, and therefore, further research needs to be conducted with a curved shape; In addition, the shear bond strength of PEEK compared to different composite resins should also be tested, as values could vary with or without wire fixation. Within the limitations of this *in vitro* study, it was concluded that FLRs made of PEEK, with a hemielliptical cross-sectional shape and a thickness-to-width ratio of 1.0:1.5, demonstrated promising results. These findings suggest that PEEK can be a viable alternative material choice for orthodontic FLRs.

## Declaration of competing interest

The authors have no conflicts of interest relevant to this article.
